# Prevalence, clinical and economic burden of mucormycosis-related hospitalizations in the United States: a retrospective study

**DOI:** 10.1186/s12879-016-2023-z

**Published:** 2016-12-01

**Authors:** Dimitrios P. Kontoyiannis, Hongbo Yang, Jinlin Song, Sneha S. Kelkar, Xi Yang, Nkechi Azie, Rachel Harrington, Alan Fan, Edward Lee, James R. Spalding

**Affiliations:** 1 Division of Internal Medicine, The University of Texas MD Anderson Cancer Center, Houston, TX USA; 2 Department of Infectious Diseases, Infection Control and Employee Health, Unit 1416, The University of Texas MD Anderson Cancer Center, 1515 Holcombe Boulevard, Houston, TX 77030 USA; 3 Analysis Group, Boston, MA USA; 4 Astellas Pharma Global Development, Inc., Northbrook, IL USA

**Keywords:** Mucormycosis, Prevalence, Clinical and economic burden

## Abstract

**Background:**

Mucormycosis is a rare but devastating fungal infection primarily affecting immunocompromised patients such as those with hematological malignancy, bone marrow and solid organ transplantation, and patients with diabetes, and, even more rarely, immunocompetent patients. The objective of this study was to assess the prevalence and burden, both clinical and economic, of mucormycosis among hospitalized patients in the U.S.

**Methods:**

This is a retrospective study using the Premier Perspective^TM^ Comparative Database, with more than 560 participating hospitals covering 104 million patients (January 2005-June 2014). All hospitalizations in the database were evaluated for the presence of mucormycosis using either an ICD-9 code of 117.7 or a positive laboratory result for Mucorales. Hospitalizations were further required to have prescriptions of amphotericin B or posaconazole to be considered as mucormycosis-related hospitalizations. The prevalence of mucormycosis-related hospitalizations among all hospital discharges was estimated. Mortality rate at discharge, length of hospital stay, and readmission rates at 1 and 3 months were evaluated among mucormycosis-related hospitalizations. Cost per hospital stay and average per diem cost (inflated to 2014 USD) were reported.

**Results:**

The prevalence of mucormycosis-related hospitalizations was estimated as 0.12 per 10,000 discharges during January 2005-June 2014. It increased to 0.16 per 10,000 discharges if the definition of mucormycosis was relaxed to not require the use of amphotericin B or posaconazole. The median length of stay was 17 days, with 23% dead at discharge; readmission rates were high, with 30 and 37% of patients readmitted within one and three months of discharge, respectively. The average cost per hospital stay was $112,419, and the average per diem cost was $4,096.

**Conclusions:**

The study provides a recent estimate of the prevalence and burden of mucormycosis among hospitalized patients. The high clinical and economic burden associated with mucormycosis highlights the importance of establishing active surveillance and optimizing prophylactic and active treatment in susceptible patients.

**Electronic supplementary material:**

The online version of this article (doi:10.1186/s12879-016-2023-z) contains supplementary material, which is available to authorized users.

## Background

Mucormycosis (formerly known as zygomycosis) is an uncommon, opportunistic fungal infection primarily caused by Mucorales, a filamentous fungus of the Mucormycetes class. This particular fungal infection typically shows a rapid progression and primarily affects patients with diabetes mellitus or compromised immune systems, such as those with hematologic malignancies, stem cell and solid organ transplants [1, 2]. Information on the prevalence of this disease is limited as there is no systematic reporting and non-specialized physicians can misdiagnose it in clinical practice. One study that used population-based active laboratory surveillance estimated that there were 1.7 mucormycosis cases per million individuals per year in the San Francisco Bay Area between 1992 and 1993 [3]. A more recent study in the U.S. reported an annual incidence rate of 0.07 and 0.29% in patients undergoing solid organ transplants and hematopoietic cell transplants, respectively, during 2001-2006 [4]. In addition, an analysis of U.S. hospital discharge data from the Healthcare Cost and Utilization Project – Nationwide Inpatient Sample (HCUP-NIS) reported 5,515 mucormycosis cases among 146 million hospitalizations of high-risk patients, i.e., immunocompromised, critically ill patients and patients with major surgery or pneumonia during 2003-2010. The number of mucormycosis cases was extrapolated to ~5,800 if all the ~ 300 million hospitalizations in the database were included for evaluation (~0.19 cases per 10,000 discharges) [5]. With the improvement in the care of critically ill and immunocompromised patients in the past few years, the prevalence of invasive fungal infections, including mucormycosis, might have changed [1, 3, 6, 7].

Amphotericin B (especially its lipid formulation), posaconazole and the recently approved triazole, isavuconazole, in conjunction with surgery, are the only active agents against mucormycosis, and are recommended by treatment guidelines [8–13]. Even with treatment, however, mucormycosis is associated with a high mortality rate [14]. Based on two systematic reviews, survival among patients treated with antifungal therapy and/or surgery ranged from 52.2 to 70% [15, 16]. A study using data from 2003-2010, reported an in-hospital mortality rate of 22% among inpatient mucormycosis cases [5].

In addition to the high mortality rate, mucormycosis is also associated with long hospital stays and substantial costs. Based on a study published in 2012, the average direct cost per episode of mucormycosis was estimated to be $113,511 (2004 USD), which was higher than more common invasive infections such as aspergillosis ($65,001; 2004 USD) and candidiasis ($81,271; 2004 USD) [17].

To provide an up-to-date overview on the epidemiology and burden of mucormycosis in the general population of hospitalized patients in U.S., we sought to estimate the prevalence of mucormycosis and to assess the clinical and economic burden associated with mucormycosis-related hospitalizations using data from recent years.

## Methods

### Data source

The study used the Premier’s Perspective™ Comparative Database (Premier), spanning from January 2005 to June 2014. Premier is a large, U.S. hospital-based database covering more than 560 participating hospitals and 104 million patients. Participating hospitals represent all regions of the U.S., are mainly non-teaching facilities, and mostly serve urban patient population. Information collected includes patient demographics (age, gender, and race/ethnicity), principal and secondary diagnoses, principal and secondary procedures, length of stay, drug utilization (drug name and strength, quantity dispensed, and unit cost), and cost of care. Laboratory results are available from the SafetySurveillor™ product from 2009 and on for about 37% of the hospitals.

### Sample selection for mucormycosis-related hospitalizations

Mucormycosis-related hospitalizations were identified as hospitalizations with an International Classification of Diseases 9th Edition (ICD-9) code of 117.7 (either primary or secondary) or a positive laboratory microbiology result for Mucorales, the organisms that cause mucormycosis [10]. To decrease the chance of false positive results, we further required all eligible encounters to have at least one prescription of amphotericin B or posaconazole to be qualified as mucormycosis-related hospitalizations. Amphotericin B and posaconazole were selected because they are the only treatments with efficacy in treating mucormycosis [8, 9, 18–21].

### Prevalence estimation

Prevalence of mucormycosis was estimated annually by dividing the number of mucormycosis-related hospitalizations by the total number of all hospital discharges observed in the same year. An alternative calculation of the prevalence was conducted by relaxing the definition of mucormycosis-related hospitalizations to require only the ICD-9 code or a positive laboratory microbiology test, without the antifungal drug use restriction.

### Analysis of clinical and economic burden

Characteristics of hospitals and patients with mucormycosis-related hospitalizations were summarized. Hospital characteristics included region, bed size, teaching facilities, and urban/rural setting. Patient characteristics included age, gender, race, and underlying conditions, and were summarized based on disease history present in the data within 12 months preceding the admission with mucormycosis.

The clinical and economic outcomes assessed included death at discharge, length of stay, and readmission rates at 1 and 3 months. Death at discharge was identified using discharge status ‘expired’. The cost for hospitalization included all supplies, labor, depreciation of equipment, etc. All costs were inflated to 2014 USD using the medical care component of the Consumer Price Index. Continuous variables were summarized by mean, standard deviation, median, and range; proportions were reported for categorical variables. All analyses were conducted using SAS 9.3.

## Results

### Prevalence

**Fig. 1:**
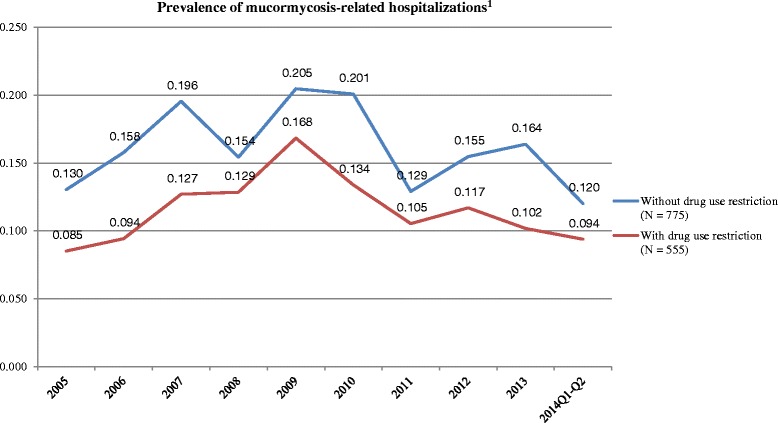
Prevalence of mucormycosis-related hospitalizations. Note: [1] Mucormycosis encounters with drug restriction were identified using ICD-9 codes or laboratory results and required at least one use of amphotericin B or posaconazole

A total of 555 mucormycosis-related hospitalizations were identified among 47,131,360 total inpatient encounters in the Premier data during January 2005 to June 2014. (Additional file 1: Figure S1) The estimated prevalence was 0.12 (range 0.09, 0.17) per 10,000 discharges during the study period. There was no clear trend of changes in prevalence across the years. In the sensitivity analysis, the estimated prevalence increased to 0.16 (range 0.12, 0.20) per 10,000 discharges (Fig. 1) after the removal of antifungal drug use restriction.

### Hospital and patient characteristics

The identified mucormycosis-related hospitalizations were from 177 distinct hospitals. Most of these hospitals were large (67%) with a bed size > 300, and 42% of the hospitals were affiliated with teaching facilities. Our sample was predominantly from urban hospitals, accounting for more than 90% of the inpatient mucormycosis encounters included in this study (Table 1).

**Table 1:** Characteristics of Hospitals and Patients

Hospital characteristics	Hospitals with Mucormycosis-related hospitalizations (*N* = 177)
*Region, n (%)*	
Midwest	32 (18)
Northeast	22 (12)
South	80 (45)
West	43 (24)
*Bed size range, n (%)*	
< 100	3 (2)
100–199	17 (10)
200–299	38 (21)
300–499	60 (34)
500+	59 (33)
*Teaching facility, n (%)*	
No	102 (58)
Yes	75 (42)
*Urban-rural setting, n (%)*	
Urban	162 (92)
Rural	15 (8)
Patient characteristics	Patients with mucormycosis-related hospitalizations (*N* = 555)
*Gender, n (%)*	
Female	205 (37)
Male	350 (63)
*Race, n (%)*	
White	341 (61)
Black	73 (13)
Hispanic	28 (5)
Other	113 (20)
*Age, years, mean (SD)*	51.7 (19.6)
*Age, years, n (%)*	
< 18	44 (8)
18–64	351 (63)
≥ 65	160 (29)
*Underlying conditions, n (%)*	
Diabetes	286 (52)
Hematological malignancies	222 (40)
Other invasive fungal infection	92 (17)
Transplant complications	89 (16)
Major solid organ transplantation	82 (15)
Stem cell transplantation	63 (11)
Solid tumor	36 (6)
Trauma/burn	20 (4)
HIV/AIDS	10 (2)

The average age of the patients with mucormycosis-related hospitalizations was 51.7 years. About 29% of them were aged 65 years or older. 63% of the patients were males and approximately 61% were white. The most common underlying condition was diabetes (52%), followed by hematological malignancies (40%) (Table 1).

### Clinical and economic outcomes

The median length of stay for the patients was approximately 17 days. The range for the length of stay was relatively wide, extending from 1 to 259 days. A total of 225 (41%) patients were admitted to intensive care units (ICUs) during the mucormycosis-related hospitalizations. The death rate at discharge was high, with 23% of the patients dead at discharge. The rate was somewhat higher among patients with hematological malignancies than those with diabetes (23 vs. 19%). Nearly one third of the patients (30%) were readmitted into hospitals within a month after the discharge, and 37% were readmitted within 3 months of the discharge (Table 2). The most common antifungal drugs used were amphotericin B lipid complex (52%), followed by liposomal amphotericin B (45%) and posaconazole (45%), which was consistent with what was observed in the sample without requiring the use of amphotericin B or posaconazole (*N* = 775). *(*Additional file 2: Table S1)

**Table 2:** Clinical and Economic Outcomes During Mucormycosis-Related Hospitalizations

Clinical outcomes	Mucormycosis-related hospitalizations (*N* = 555)
*Length of stay in days, Median (range)*	17 (1–259)
*Deaths at discharge, n (%)*	130 (23)
*1-month readmission, n (%)*	168 (30)
*3-month readmission, n (%)*	206 (37)
Economic outcomes	Cost (2014 USD)
*Total cost per hospital stay*	
Mean (SD)	$112,419 ($159,144)
Median (Range)	$57,324 ($1,912 - $1,395,163)
*Per diem cost*	
Mean (SD)	$4,096 ($2,683)
Median (Range)	$3,442 ($768 - $19,728)

The average cost per hospital stay was $112,419 (standard deviation $159,144) with a range of $1,912 to $1,395,163. The mean per diem cost during hospitalization was $4,096, with a range of $768 to $19,728 (Table 2). The mean daily cost for patients with hematologic malignancies was $4,343 (standard deviation $2,733) with a range of $1,081 to $19,728), while it was $3,761 (standard deviation $2,327) with a range $768 to $16,387 for those with diabetes. The mean daily cost for patients with and without ICU admission during the hospitalization was $5,107 (standard deviation $2,995) and $3,407 (standard deviation $2,200), respectively.

## Discussion

The current study utilized recent hospital-based data, ranging from January 2005 to June 2014, and found that the prevalence of mucormycosis-related hospitalizations during this period was 0.12 per 10,000 discharges. The estimate was lower than what has been reported in the 2003-2010 HCUP-NIS study for two possible reasons [5]. Firstly, the current study utilized more recent data (January 2005 to June 2014) and therefore reflected the prevalence of a different time period than the HCUP-NIS study. More importantly, the current study required the use of amphotericin B or posaconazole when identifying mucormycosis-related hospitalizations to reduce false positives, which has been previously used and validated by a number of studies [22]. We noticed that 28% of hospital stays with an ICD-9 code or microbiology results of mucormycosis in our study did not have any prescriptions for amphotericin B or posaconazole. Hence, using only ICD-9 code to estimate prevalence may potentially lead to an over-estimate of the prevalence. Both the median length of hospital stay observed and the average cost per hospital stay in our study were greater than those in the HCUP-NIS study (17 days vs. 14 days and $112,419 vs. $90,107, respectively) [5], which can also be due to the potential inclusion of false positives by using only ICD-9 code when identifying mucormycosis-related hospitalizations.

When the definition for mucormycosis-related hospitalizations was relaxed to not require anti-fungal use, the overall prevalence increased to 0.16 per 10,000 discharges, which was similar to that reported in the 2003-2010 HCUP-NIS study. This is a 23% increase in prevalence when compared to 0.13 per 10,000 discharges reported for the period of 1992-1993 by Rees et al., (which also did not require anti-fungal drug use when identifying mucormycosis-related hospitalizations) [3]. There was, however, no clear trend observed in terms of how the prevalence has changed between 2005 and 2014 from the present study.

In our study, the economic burden associated with a mucormycosis-related hospitalization was substantial, with average cost per stay at $112,419, which was comparable to the cost of mucormycosis reported in the literature ($113,511; 2004 USD), and higher than the average cost reported for both aspergillosis-related hospitalizations and candidiasis-related hospitalizations ($65,001 and $81,271; 2004 USD, respectively), reflecting the significant morbidity and mortality of mucormycosis [17]. Specifically, the high cost per mucormycosis-related hospitalizations could be related to patients’ prolonged hospital stays, heavy use of intensive care services, and often severe underlying conditions [5]. By multiplying the prevalence of mucormycosis and the average cost of mucormycosis-related hospitalizations observed in this study with the total number of US hospital discharges in 2013 (35,597,792), we projected that the total cost associated with mucormycosis could amount to approximately $48 million per year.

Amphotericin B lipid complex (52%) was the most commonly used treatment, followed by liposomal amphotericin B (45%) and posaconazole (45%) among patients with mucormycosis-related hospitalizations observed in the present study. Although Mucorales have been shown to be resistant to drugs such as fluconazole and voriconazole, they were used in 21% and 23% of the mucormycosis-related hospitalizations. Due to lack of time stamps of the drug prescriptions, it was not clear if these fluconazole and voriconazole prescriptions were intended to treat mucormycosis or were intended to serve as prophylaxis treatment for patients with high risk of various invasive fungal infections, such as patients with leukemia and recipients of stem cell transplantation [8, 23]. Given the study period (2005 – 2014), data regarding the more recently approved drug isavuconazole were not captured.

Our study has several limitations that should be taken into consideration when interpreting the results. First, only a subset of hospitals in Premier submitted laboratory microbiology results, and laboratory microbiology results were not available before 2009. In addition, it is possible that some of the hospitalizations without use of amphotericin B or posaconazole were true mucormycosis cases, or that some mucormycosis cases were identified post-mortem, which might not have been completely captured by the data [24, 25]. Due to lack of histopathology data, cases identified by histopathology only and didn’t have positive microbiology results or the ICD-9 code could have been missed as well. Such limitations coupled with a high rate of underdiagnoses for mucormycosis in clinical practice could have led to an underestimation of the prevalence of mucormycosis in our study [8, 26]. Second, more than 90% of the encounters included in the final sample were from urban hospitals, which might not be representative of U.S. hospitals. Third, clinically important information regarding the presentation of mucormycosis such as sites of involvement and infecting Mucorales species was not captured in the Premiere Database. Finally, the costs of mucormycosis-related hospitalizations varied significantly, which could be due to the different underlying conditions associated with the mucormycosis-related hospitalizations [27]. Further studies identifying the additional cost attributable solely to mucormycosis are needed to provide more accurate estimates of the burden of mucormycosis.

## Conclusions

The low prevalence of mucormycosis, its high clinical and economic burden, and the fact that commonly used antifungal therapies such as fluconazole and voriconazole are not effective against mucormycosis, indicate a need to increase the awareness of mucormycosis among physicians who routinely treat patients at high risk mucormycosis for the infection, and the necessity of active surveillance and optimized prophylactic treatment in susceptible individuals.

## Additional files

Additional file 1: Figure S1. Sample Selection Flow Chart of Mucormycosis-Related Hospitalizations. (TIF 707 kb)
Additional file 2: Table S1. Antifungal Drug Use. Presents the prevalence of antifungal drug uses during the mucormycosis-related hospitalizations with and without requiring use of amphotericin B or posaconazole. (DOCX 29 kb)

## Abbreviations

HCUP-NIS: Healthcare Cost and Utilization Project – Nationwide Inpatient Sample
ICD-9: International Classification of Diseases 9th Edition
SD: Standard deviation
