# Supplementary figures and images for: Prevalence, clinical and economic burden of mucormycosis-related hospitalizations in the United States: a retrospective study
Source: BMC Infect Dis. 2016 Dec 1;16:730. doi: 10.1186/s12879-016-2023-z (PMC5134281; doi:10.1186/s12879-016-2023-z)

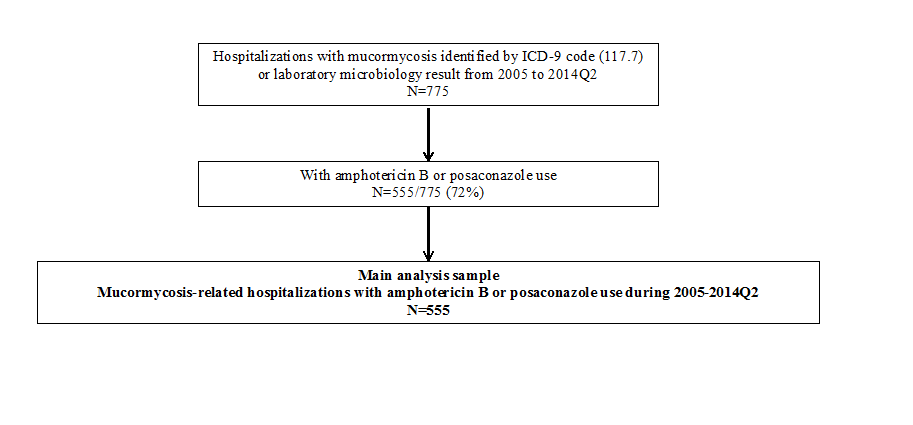

Supplement: Additional file 1: Figure S1. — Sample Selection Flow Chart of Mucormycosis-Related Hospitalizations. (TIF 707 kb) [file 12879_2016_2023_MOESM1_ESM.tif]
