# Supplementary material for: Prevalence, clinical and economic burden of mucormycosis-related hospitalizations in the United States: a retrospective study
Source: BMC Infect Dis. 2016 Dec 1;16:730. doi: 10.1186/s12879-016-2023-z (PMC5134281; doi:10.1186/s12879-016-2023-z)
Supplement: Additional file 2: Table S1. — Antifungal Drug Use. Presents the prevalence of antifungal drug uses during the mucormycosis-related hospitalizations with and without requiring use of amphotericin B or posaconazole. (DOCX 29 kb) [file 12879_2016_2023_MOESM2_ESM.docx]

| **Supplementary Table 1. Antifungal Drug Use** | |  |
| --- | --- | --- |
| **Antifungal drug use** | **Mucormycosis-related hospitalizations with anti-fungal drug use restriction**  **(N = 555)** | **Mucormycosis-related hospitalizations without anti-fungal drug use restriction**  **(N = 775)** |
| *Amphotericin B compounds/polyenes, n (%)* | |  |
| Conventional Amphotericin B | 81 (15) | 81 (10) |
| Amphotericin B lipid complex | 287 (52) | 287 (37) |
| Amphotericin B colloidal dispersion | 0 (0) | 0 (0) |
| Amphotericin B liposome | 249 (45) | 249 (32) |
| *First Generation Azoles, n (%)* |  |  |
| Fluconazole | 118 (21) | 146 (19) |
| Itraconazole | 6 (1) | 9 (1) |
| *Second Generation Azoles* |  |  |
| Voriconazole | 129 (23) | 151 (19) |
| Posaconazole | 248 (45) | 248 (32) |
| *Echinocandins, n (%)* |  |  |
| Caspofungin | 76 (14) | 83 (11) |
| Micafungin | 59 (11) | 70 (9) |
| Anidulafungin | 5 (1) | 5 (1) |
|  |  |  |
| *Flucytosine, n (%)* | 3 (1) | 3 (0) |
